# Radiofrequency venous ablation for symptomatic relief in postural orthostatic tachycardia syndrome: a case series

**DOI:** 10.1093/ehjcr/ytae029

**Published:** 2024-01-23

**Authors:** Catherine L B McGeoch, Rebecca S Steinberg, Kristina S Bortfeld, Zakaria Almuwaqqat, J Mark Rheudasil, Neal K Bhatia, Alexis C Cutchins

**Affiliations:** Department of Medicine, Emory University School of Medicine, Atlanta, GA, USA; Department of Medicine, Emory University School of Medicine, Atlanta, GA, USA; Department of Medicine, Emory University School of Medicine, Atlanta, GA, USA; Department of Medicine, Division of Cardiology, Emory University School of Medicine, Atlanta, GA, USA; Department of Surgery, Division of Vascular Surgery and Endovascular Surgery, Emory University School of Medicine, Atlanta, GA, USA; Department of Medicine, Division of Cardiology, Emory University School of Medicine, Atlanta, GA, USA; Department of Medicine, Division of Cardiology, Emory University School of Medicine, Atlanta, GA, USA

**Keywords:** Postural orthostatic tachycardia syndrome, Pelvic congestion syndrome, Venous ablation, Hypovolemia, Radiofrequency ablation, Case series

## Abstract

**Background:**

Hypovolemic postural orthostatic tachycardia syndrome (POTS) is thought to be caused by dysregulated circulating blood volume. Management is mainly limited to symptom-targeted lifestyle changes. Radiofrequency venous ablation (RFA) represents a minimally invasive method of increasing circulating blood volume. The following case series describes a novel application of RFA to successfully target POTS symptoms in patients demonstrating venous insufficiency. The use of RFA in alleviating POTS symptoms has not previously been reported.

**Case summary:**

We describe four patients with either a well-established historical POTS diagnosis or dysautonomia symptoms refractory to both medical management and lifestyle modifications. They all demonstrated venous reflux on lower extremity venous ultrasound testing. Upon vascular surgery referral, all underwent great and small saphenous vein RFA. They each subsequently reported subjective improvement in their dysautonomia symptoms and quality-of-life. Two with symptom recurrence years later were found to have new-onset pelvic venous congestion and are being evaluated for pelvic venous insufficiency interventions.

**Discussion:**

Lower extremity venous pooling can exacerbate dysautonomia symptoms in POTS patients. Patients refractory to conventional treatment strategies should undergo venous insufficiency workup, and if positive, should be referred for venous pooling intervention evaluation. The success of RFA at treating refractory POTS symptoms in these four patients with lower extremity venous reflux, including no surgical intervention and no adverse effects, are compelling grounds to further explore this therapy and to quantify and standardize symptom improvement assessment in a larger patient population. Future directions include a demonstration of quality-of-life improvement in randomized clinical trials.

**Learning points**

- Patients with postural orthostatic tachycardia syndrome (POTS) may experience symptom exacerbation due to lower extremity venous insufficiency and/or pelvic venous congestion, and those refractory to conventional treatment strategies should undergo venous insufficiency evaluation, and if positive, evaluation for minimally invasive radiofrequency ablation (RFA).
- RFA of the great and small saphenous veins should be considered as a safe, novel treatment to alleviate a variety of POTS symptoms and improve quality-of-life in this patient population.

## Introduction

An estimated 500 000 to 3 million Americans suffer from postural orthostatic tachycardia syndrome (POTS).^1^ European prevalence data remains largely unavailable.^2^ Formal diagnostic criteria are classically defined as chronic orthostatic intolerance and a ≥30 beats/min increase in heart rate upon standing without orthostatic hypotension.^3^ Patients commonly present with complaints of the lower extremity (LE) pain and swelling, palpitations, fatigue, nausea, ‘brain fog’, lightheadedness, exercise intolerance, tremulousness, and syncope. It is important to note that not all patients seeking POTS treatment have a record of these formal diagnostic criteria, yet the following cases demonstrate that these patients still benefit from the same symptomatic workup as those who do. Management of POTS can be challenging for both patients and providers, and treatment strategies have been overall inconsistent.

## Summary figure

**Table:** 

Case number	Age		Lower extremity venous duplex exam findings	Right leg CEAP before ablation	Left leg CEAP before ablation	POTS symptoms before ablation	POTS symptoms improved after ablation
1	37	POTS diagnosis 5 years before initial visit, chronic Lyme disease, portal and superficial vein thrombosis, Lupus	Bilateral deep and superficial venous reflux (common femoral, femoral, sapheno-femoral, great saphenous)	CEAP^a^: 3	CEAP: 3	Diaphoresis, fatigue, lower extremity oedema and pain, palpations, dizziness	Decreased lower extremity oedema and fatigue, increased exercise tolerance
2	54	Obstructive sleep apnoea, hypothyroidism, autonomic dysfunction, varicose veins	Bilateral great and small saphenous reflux (small and great saphenous) + pelvic venous insufficiency	CEAP: 1	CEAP: 1	Sensation of a racing and audible heartbeat while lying down, frequent falls	‘Everything has stabilized’, ‘feeling much better’
3	29	Sinus sick syndrome	Diffuse bilateral venous reflux (common femoral, sapheno-femoral, proximal great saphenous, sapheno-popliteal)	CEAP: 3	CEAP: 3	Dizziness, tinnitus, palpitations, sensations of a rapid heartbeat	Resolved dizziness and tinnitus; ‘feeling much better’
4	25	Childhood POTS	Bilateral great and small saphenous reflux (common femoral, sapheno-femoral, proximal great saphenous, small saphenous)	Not provided	Not provided	Fatigue, dyspnoea, shooting pain down lower extremities, chest pain, rapid heartbeat, increased anxiety	‘Doing beautifully’, ‘dramatic symptom improvement’

^a^CEAP = clinical-etiology-anatomy-pathophysiology classification of venous disorders; based on visible skin changes; ranges from 0 (no venous disease) to 6 (open and active ulcer).

Postural orthostatic tachycardia syndrome is a physiologically complex disorder with poorly understood pathophysiology. Proposed mechanisms include autonomic dysfunction, venous pooling, and connective tissue disorders. Blood volume dysregulation is a well-established mechanism of primary POTS.^4^ As circulating blood volume decreases, arterial baroreceptors respond by increasing sympathetic activation and elevating heart rate, with worsening of symptoms upon standing. Patients with hypovolemic POTS may increase circulating blood volume through exercise, hydration, salt loading, and compression stockings. These patients are often prescribed fludrocortisone and desmopressin in an effort to retain water and salt and to limit potential fluctuations in circulating blood volume.

Other therapies are limited; however, treatment of venous insufficiency or pelvic venous congestion (PVC) may limit venous pooling and increase circulating blood volume. Venous ablation is a minimally invasive, safe, and effective procedure indicated for the treatment of varicose veins and symptomatic varicosities such as oedema, aching sensations, bruising, and itching.^5^ Radiofrequency ablation (RFA) in particular is indicated for incompetent great saphenous veins (GSV) and small saphenous veins (SSV) and has shown similar clinical outcomes to endovenous laser ablation.^6^ Its use in alleviating POTS symptoms has not previously been reported. We describe four patient presentations that demonstrated marked improvement after RFA for their POTS symptoms.

## RFA technique

**Figure 1:**
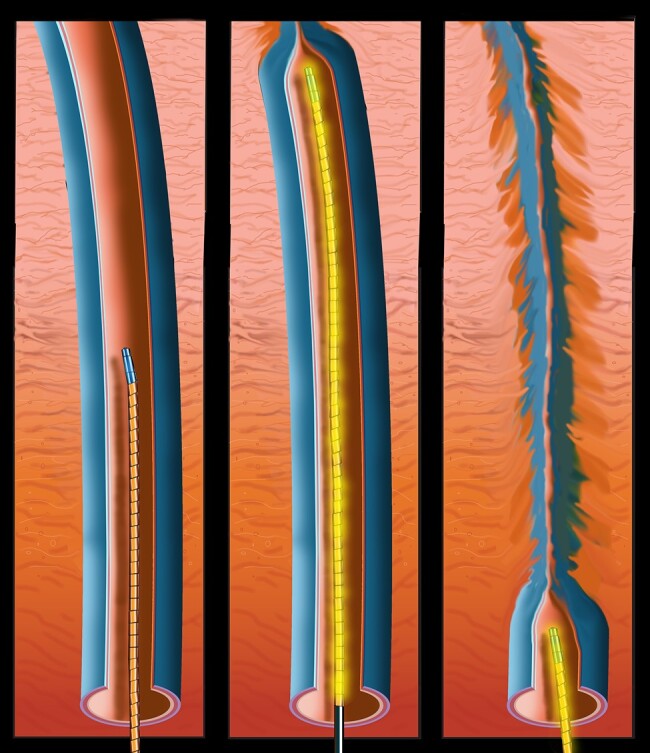
Radiofrequency ablation rendering with a generic catheter. Left panel: catheter insertion. Middle panel: controlled radiofrequency heat energy applied. Right panel: heat causes vein collapse. Illustration by Sebastian Keneas.

A generic RFA representation is illustrated in *Figure 1*. Radiofrequency energy (heat) is applied via a catheter placed in the diseased vein of interest. The controlled heat collapses the vein so that it closes when the catheter is removed. While there are multiple techniques for vein ablation, the one that our institution uses in the majority of cases is the Medtronic Closurefast RFA system.

## Case 1

A 37-year-old woman with a history of chronic Lyme disease, portal and superficial vein thrombosis, and Lupus presented to our clinic 5 years after her initial POTS diagnosis. Her POTS symptoms (diaphoresis, fatigue, LE oedema and pain, palpitations, and dizziness) began at age 12 when she was diagnosed with Lyme disease. She tried metoprolol, prednisone, midodrine, omalizumab, compression stockings, oral hydration, gluten-free diet, salt supplementation, and exercise. She was referred for RFA when her leg swelling persisted with conservative management and pharmacotherapy. On physical exam, she had moderate varicosities over her bilateral posteromedial calves with 1+ oedema. She underwent bilateral GSV and left SSV ablation. Post-RFA, she reported improvements in LE oedema, exercise intolerance, and fatigue. Two years later, she reported increased oedema, weight gain, and recurrence of her POTS symptoms, and was discovered to have iliac vein obstruction. She is undergoing workup for pelvic vein intervention.

## Case 2

A 54-year-old woman with a history of obstructive sleep apnoea, hypothyroidism, autonomic dysfunction, and varicose veins (noted to be severe internally during a hysterectomy) presented thirteen years after the onset of her autonomic symptoms—initially, a sensation of a racing and audible heartbeat when supine, and several years later, frequent falls. She was diagnosed with POTS and started on carvedilol, which was later switched to propranolol and clonazepam. On physical exam, she was noted to have bilateral LE varicose veins. Renal ultrasound showed potential fibromuscular dysplasia, and LE venous Doppler showed bilateral venous insufficiency. Vein clinic recommended staged bilateral GSV/SSV RFA, but she opted for further conservative management. She tried increasing exercise and salt intake, compression to the abdomen, loratadine, famotidine, and nadolol with modest symptom improvement. She decided to undergo bilateral GSV and SSV ablation. Afterward, she reported resolved leg pain, improved tremors, and endorsed feeling better, stating that she feels ‘much better’ and that ‘everything has stabilized.’ Improvements have lasted for almost two years to date post-ablation.

## Case 3

A 29-year-old woman with a history of sinus sick syndrome status-post permanent pacemaker placement presented to our POTS clinic. Symptoms included dizziness when bending down, tinnitus, palpitations, and sensations of a rapid heartbeat. She tried compression leggings, increasing water intake, and electrolyte supplementation with no symptom improvement. Our clinic started her on nadolol, which initially helped, but dizziness and worsening tinnitus recurred, and she experienced new-onset ankle swelling despite wearing compression pants, dyspnoea upon moving from lying down to sitting up, and palpitations. Three months after restarting nadolol, she felt like she would pass out upon standing, and our clinic switched her nadolol to acebutolol, added a clonidine patch, and started fludrocortisone to address episodes of hypotension. LE venous Doppler studies were positive for reflux. After 7 months of minimal symptom improvement, she underwent bilateral GSV and SSV RFA. Through 1-year post-RFA, she reported feeling ‘much better’ with improved dizziness and tinnitus. This symptomatic improvement began within a few weeks of her ablation. Thirteen months post-RFA, her remaining symptom was lightheadedness, with new common femoral vein reflux and iliac vein compression discovered on repeat bilateral LE venous Doppler, and she is considering further venous insufficiency intervention. At nearly 2 years of follow-up to date, she has noted persistent symptom improvement of her most distressing symptom, fainting, commenting that before her ablation, she would often become dizzy and pass out but that since her ablation she has not passed out.

## Case 4

A 25-year-old woman with a history of POTS presented for management of symptom recurrence. She was initially diagnosed with POTS at age 12 following a mononucleosis infection. At age 16, symptoms suddenly disappeared until recurrence following COVID-19 infection 5 months before her visit to our clinic. Her symptoms included fatigue, dyspnoea, shooting LE pain, chest pain, rapid heartbeat, and anxiety, all of which inhibited her ability to go to work. She exhibited POTS symptoms during her office visit with a significant increase in heart rate from supine to standing despite sufficient hydration and compression socks. Venous Doppler studies showed bilateral GSV and SSV insufficiency, and she underwent bilateral GSV and SSV RFA. She reported ‘doing beautifully’ after the left ablation and has reported ‘dramatic symptom improvement’ since having both legs done. She commented that symptom improvement post-ablation was almost immediate—she felt like her dyspnoea improved and that her brain fog dissipated within a week of treatment. At almost 2 years of follow-up to date post-ablation, she reported feeling ‘like a new person and grateful to now be able to consistently make it to work, which was not the case before.’

## Discussion

This is the first case series to present RFA as a POTS therapy. In our robust practice of seeing POTS patients, patients without venous insufficiency are rare. This is an important finding for practitioners taking care of POTS patients to recognize and treat if needed. All four of our patients experienced meaningful symptom improvement following RFA, before which medications and lifestyle modifications had been unsuccessful.

According to the 2022 Society for Vascular Surgery clinical practice guidelines, the diagnostic test of choice for patients with LE chronic venous disease is duplex ultrasound to evaluate for venous reflux.^7^ Both splanchnic and peripheral venous pooling have been documented as a prevalent symptom in POTS patients. Compared to matched controls, POTS patients have increased resting superior mesenteric artery (SMA) blood flow and SMA time-averaged velocity as well as decreased SMA vascular resistance.^8^ One mechanistic theory is that peripheral sympathetic nerve dysfunction, predominantly in the lower limbs, leads to impaired peripheral vasoconstriction, and subsequent pooling.^9^ A recent study assessing the prevalence of venous insufficiency in POTS patients found significant left common iliac vein compression in a majority of POTS patients, with 69% prevalence among females with POTS compared to 40% among controls, suggesting that POTS patients with PVC symptoms should be assessed for venous outflow obstruction.^10^ Recently, Ormiston *et al.*^11^ published improvement in POTS symptoms following left common iliac vein stenting. The minimally invasive approaches of RFA and endovenous laser ablation show promise for improvement in POTS symptoms with reduced morbidity and recovery time, particularly in more superficial veins.^12^

Patients with PVC also develop LE superficial venous insufficiency and may have more proximal obstruction. In fact, two of the four patients discussed in this case report have documented iliac vein compression that was discovered after their LE venous ablations occurred (likely also implicated in their post-ablation symptom recurrence). Furthermore, as much as 70% of varicose veins, a dermatological manifestation of POTS which several of the patients in this case series experienced, are due to sapheno-femoral junction incompetence with reflux in the long saphenous vein.^12^ Further research into the associations between LE venous insufficiency, PVC, and POTS is clearly needed. The success of RFA at treating refractory POTS symptoms in these four patients with LE venous reflux, including no adverse effects, are compelling grounds to further explore this therapy and to quantify and standardize symptom improvement assessment in a larger patient population. Future directions include a demonstration of quality-of-life improvement in larger observational cohorts and in randomized clinical trials, which would directly assess any potential placebo effect that can accompany invasive treatment.

## Limitations

It is important to note that, as this is a preliminary, retrospective series born out of trends observed clinically, data on heart rate variation before and after RFA could not be reported. Without an objective measure of symptom improvement, a placebo effect cannot be ruled out. We look forward to conducting a much larger follow-up prospective evaluation to accompany the subjective symptom improvement patients have reported post-ablation.

## Conclusions

We present the first case series to discuss radiofrequency venous ablation as a potential therapeutic strategy in hypovolemic POTS patients with refractory symptoms. Our patients independently reported improvements in multiple symptom domains. This safe, often elective procedure has the potential to improve quality-of-life in this patient population and warrants further longitudinal investigation.

## Lead author biography


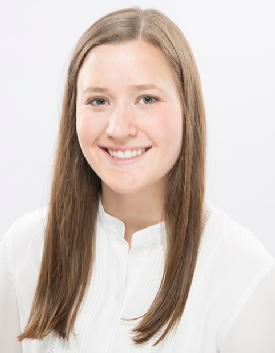
Catherine L.B. McGeoch graduated from Yale University with a B.S. in Chemistry and is now in her fourth year of medical school at Emory University. Her research focus is cardiovascular disease and prevention, particularly in women. She looks forward to her career as a physician-scientist.

## Data Availability

The data underlying this article will be shared on reasonable request to the corresponding author.
